# Loss of consumers constrains phenotypic evolution in the resulting food web

**DOI:** 10.1002/evl3.170

**Published:** 2020-04-20

**Authors:** Matthew A. Barbour, Christopher J. Greyson‐Gaito, Arezoo Sotoodeh, Brendan Locke, Jordi Bascompte

**Affiliations:** ^1^ Department of Evolutionary Biology and Environmental Studies University of Zurich Zurich 8057 ZH Switzerland; ^2^ Department of Zoology University of British Columbia Vancouver BC V6T 1Z4 Canada; ^3^ Department of Integrative Biology University of Guelph Guelph ON N1G 2W1 Canada; ^4^ Department of Biological Sciences Humboldt State University Arcata California 95521

**Keywords:** Adaptive landscape, community context, eco‐evolutionary dynamics, ecological networks, extinction, fitness landscape, host–parasitoid, natural selection

## Abstract

The loss of biodiversity is altering the structure of ecological networks; however, we are currently in a poor position to predict how these altered communities will affect the evolution of remaining populations. Theory on fitness landscapes provides a framework for predicting how selection alters the evolutionary trajectory and adaptive potential of populations, but often treats the network of interacting populations as a “black box.” Here, we integrate ecological networks and fitness landscapes to examine how changes in food‐web structure shape phenotypic evolution. We conducted a field experiment that removed a guild of larval parasitoids that imposed direct and indirect selection pressures on an insect herbivore. We then measured herbivore survival as a function of three key phenotypic traits to estimate directional, quadratic, and correlational selection gradients in each treatment. We used these selection gradients to characterize the slope and curvature of the fitness landscape to understand the direct and indirect effects of consumer loss on phenotypic evolution. We found that the number of traits under directional selection increased with the removal of larval parasitoids, indicating evolution was more constrained toward a specific combination of traits. Similarly, we found that the removal of larval parasitoids altered the curvature of the fitness landscape in such a way that tended to decrease the evolvability of the traits we measured in the next generation. Our results suggest that the loss of trophic interactions can impose greater constraints on phenotypic evolution. This indicates that the simplification of ecological communities may constrain the adaptive potential of remaining populations to future environmental change.

**Impact Summary**

The loss of biodiversity is rewiring the web of life; however, it is uncertain how this will affect the ability of remaining populations to evolve and adapt to future environments. To gain insight into these effects, we conducted a field experiment that either maintained a natural community of predators or removed all but one of the predators that was able to impose selection on a common prey. We found that the loss of predators acted to constrain prey evolution toward a particular combination of traits. Moreover, we found that the loss of predators could make it more difficult for prey to adapt to uncertain future environments. Taken together, our results suggest that the simplification of the web of life may constrain the adaptive potential of remaining populations.

The fitness landscape provides a powerful framework for understanding how natural selection has shaped the evolution of biodiversity—from genes to phenotypes to species (Wright 1931; Simpson 1944; Arnold et al. 2001). More than a metaphor, the fitness landscape links quantitative genetic and phenotypic variation in multiple traits to evolution by natural selection (Lande 1979; Arnold and Wade 1984a,b). Ecological interactions often play a key role in shaping natural selection, as evidenced by the role of antagonistic and mutualistic interactions in driving evolutionary change (Schluter 2000; Abrams 2000; Bronstein et al. 2006). Although there is clear evidence that species interactions can shape the fitness landscape, we still have a poor understanding of how the fitness landscape is shaped by a community of interacting species (McPeek 2017; Hui et al. 2018; terHorst et al. 2018). Resolution on how change in ecological communities shape phenotypic evolution is urgently needed though, given the rapid losses of biodiversity we are observing in the Anthropocene (Scheffers et al. 2016).

Ecological networks, such as food webs describing who‐eats‐whom, provide an explicit representation of the direct and indirect effects that emerge in a community of interacting species (Bascompte and Jordano 2014; McCann 2012). Here, we integrate ecological networks and fitness landscapes to understand how selection imposed by ecological communities alter the evolutionary trajectory and adaptive potential of interacting populations (Hui et al. 2018). The effects of natural selection on multiple phenotypic traits can be inferred by quantifying the slope and curvature of the fitness landscape (Arnold 1992). The slope is determined by the strength of directional selection acting on each trait, which influences the trajectory of evolution (Lande 1979; Arnold 1992). This fact is made clear by the “Lande equation,” $\Delta \bar{z}=G\beta$, where $\Delta \bar{z}$ is a column vector of the average change in each trait between generations, G is the additive genetic (co)variance matrix for these traits (i.e., G‐matrix), and β is a column vector of directional selection gradients acting on each trait (i.e., slope). Grant and Grant (1995) used the slope of the fitness landscape for multiple traits related to body and beak size in Darwin's finches to accurately predict the effects of drought on the evolutionary trajectory of these traits. The curvature of the fitness landscape is governed by the strength of stabilizing, disruptive, and correlational selection acting on each trait, which can alter the adaptive potential of a population through its effect on the G‐matrix (Hansen and Houle 2008). For example, stabilizing selection acts to erode genetic variance in a trait, which can impose a constraint on the ability of this trait to respond to future selection (Hansen and Houle 2008). In contrast, disruptive selection toward extreme trait values acts to increase genetic variance in a trait, thus increasing the capacity for future adaptation (Bolnick and Lau 2008). Correlational selection alters the genetic covariance between traits, which may facilitate or hinder future adaptation depending on the pattern of selection on those traits and the structure of the G‐matrix (Brodie 1992). If we want to predict how ecological communities shape phenotypic evolution, we must understand how ecological networks shape the fitness landscape of interacting populations.

The loss of biodiversity is altering the structure of ecological networks (Landi et al. 2018a), which may influence the slope and curvature of the fitness landscape in a number of ways. For example, consider how changes in consumer diversity in a food web may alter the slope of the fitness landscape for a shared resource. If different consumers impose directional selection on different traits of the resource, then a more diverse consumer community would increase the number of traits under selection, which may constrain the trajectory of evolution toward a specific phenotype. Alternatively, if consumers impose selection on different values of a trait, then their selective effects would cancel each other out in more diverse communities, which would allow for a greater diversity of phenotypes to persist. Now consider the effects of consumer diversity on the curvature of the fitness landscape. If consumers impose selection on different ends of a resource's trait distribution, then a more diverse consumer community may impose stabilizing selection, which would decrease genetic variance in this trait. In contrast, additional consumers may impose disruptive selection if their cumulative effect decreases the relative fitness of a resource's average trait value, which would increase the genetic variance in this trait.

To examine these different possibilities, we conducted a field experiment that removed a consumer guild that parasitizes an abundant insect herbivore (*Iteomyia salicisverruca*, Family Cecidomyiidae; Fig. 1). The larvae of this herbivore induce tooth‐shaped galls when they feed on the developing leaves of willow trees (*Salix* sp., Russo 2006). These galls protect larva from attack by generalist predators (e.g., ants, spiders), but they suffer high mortality from egg and larval parasitoids (Barbour et al. 2016). We manipulated food‐web structure by either removing larval parasitoids (removal food web) or allowing both egg and larval parasitoids to impose selection on gall midge traits (original food web, Fig. 1). Larval parasitoids also impose indirect effects on gall midge fitness through intraguild predation on the egg parasitoid (Fig. 1). We applied modern statistical methods to quantify how changes in food‐web structure altered the slope and curvature of the gall midge's fitness landscape. Taken together, our study gives insight to how the loss of biodiversity may alter both the evolutionary trajectory and adaptive potential of interacting populations.

**Figure 1:**
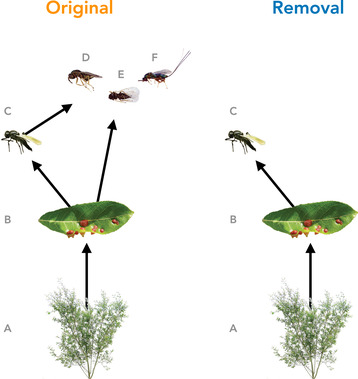
Experimental manipulation of food‐web structure associated with a leaf‐galling midge (B, *Iteomyia salicisverruca*) feeding on the willow *Salix hookeriana* (A). Black arrows denote the flow of energy in this network of trophic interactions. In the original food web, we allowed the full suite of egg and larval parasitoids to impose selection. In the removal food web, we used mesh bags to exclude the guild of larval parasitoids, only allowing the egg parasitoid (C, *Platygaster* sp.) to impose selection. Note that larval parasitoids also impose indirect effects on gall midge fitness through intraguild predation on the egg parasitoid. Larval parasitoids include the following species: *Mesopolobus* sp. (D, Family: Pteromalidae); *Tetrastichus* sp. (E, Family: Eulophidae); and *Torymus* sp. (F, Family: Torymidae).

## Methods

### STUDY SITE

We conducted our study within a four‐year old common garden experiment of coastal willow (*Salix hookeriana*) located at Humboldt Bay National Wildlife Refuge (HBNWR) (40, 40${}^{\prime }$53${}^{\prime \prime }$N; 124 12${}^{\prime }$4${}^{\prime \prime }$W) near Loleta, California, USA. This common garden consists of 26 different willow genotypes that were collected from a single population of willows growing around Humboldt Bay. Stem cuttings of each genotype (25 replicates per genotype) were planted in a completely randomized design in two hectares of a former cattle pasture at HBNWR. Willows at our study site begin flowering in February and reach their peak growth in early August. During this study, willows had reached 5–9 m in height. Further details on the genotyping and planting of the common garden are available in Barbour et al. (2015).

### MANIPULATING FOOD‐WEB STRUCTURE

We setup our food‐web manipulation across 128 plants soon after galls began developing on willows in early June of 2013. These 128 plants came from 8 different plant genotypes that spanned the range of trait variation observed in this willow population (Barbour et al. 2015). For the original food web (eight replicates per genotype), we used flagging tape to mark 14 galled leaves per plant (~30 larvae), allowing the full suite of egg and larval parasitoids to impose selection. Marking galls with flagging tape ensured that we compared galls with similar phenology in both treatments when we collected galls later in the season. For the removal food web, we enclosed 14 galled leaves with 10×15 cm organza bags (ULINE, Pleasant Prairie, WI, USA) to exclude three parasitoid species that attack during larval development. This treatment did not exclude the egg parasitoid *Platygaster* sp., which attacks prior to gall initiation (larva initiate gall development in Cecidomyiid midges: Gagné 1989). It was not possible to reciprocally manipulate parasitoid attack (i.e., exclude egg parasitoid, but allow larval parasitoids) because it was not possible to identify midge oviposition sites prior to gall formation. In late August, we collected marked and bagged galls from each plant, placed them into 30 mL vials and kept them in the lab for four months at room temperature. We then opened galls under a dissecting scope and determined whether larvae survived to pupation (our measure of fitness) or died due to parasitism. We further distinguished whether mortality was caused by an egg or larval parasitoid. Early larval death was another important source of mortality (17%), but we excluded these cases from our analysis. We did this because early larval death can stunt gall growth, which would confound estimates of selection due to parasitism on one of the phenotypes we measured (chamber diameter; details given in next section). For the food‐web treatment that excluded larval parasitoids, we further restricted our data by removing any incidental instances of parasitism by a larval parasitoid. This represented less than 3% of the observations in this food‐web treatment and allowed us to focus our inferences of selection on those imposed by the egg parasitoid. Our final dataset contains survival estimates for 1285 larvae from 613 galls and 111 plants.

### MEASURING PHENOTYPIC TRAITS

We collected data on three different traits that we expected to influence larval survival based on previous work in this system (Barbour et al. 2016) and other work with gall midges (Weis et al. 1983; Heath et al. 2018). First, we measured gall diameter as the size of each gall chamber to the nearest 0.01 mm at its maximum diameter (perpendicular to the direction of plant tissue growth). Previous work in this system has shown that larger galls are associated with higher survival (Barbour et al. 2016). Second, we measured clutch size by counting the number of chambers in each gall (Weis et al. 1983; Heath et al. 2018). All larvae collected from the same multichambered gall were scored with the same clutch size. Third, we measured oviposition preference for individual plants as the density of larvae observed on a plant in an independent survey. We did this by randomly sampling five branches per tree and counting the number of individual gall chambers (number of larvae). We then converted these counts to a measure of larval density per 100 shoots by counting the number of shoots on the last branch we sampled. All larvae collected from the same plant were scored with the same oviposition preference. Measuring larval densities on plants in the field is a common method for measuring oviposition preference (Gripenberg et al. 2010); however, caution must be taken in inferring “preference” as larval densities can be influenced by processes other than preference (Singer 1986). Fortunately, two features of our study system suggest that larval densities may be a good proxy for oviposition preference. First, because our data come from a randomized placement of plant genotypes in a common garden, female midges have equal exposure to many possible plant genotypes when choosing an oviposition site. Second, egg predation is a minor source of mortality for galling insects in general (Hawkins et al. 1997); therefore, we do not expect any prior egg predation to bias our estimates of observed larval densities.

### QUANTIFYING THE FITNESS LANDSCAPE

Inferring the fitness landscape assumes that trait distributions are multivariate normal (Lande and Arnold 1983). To approximate this assumption, we log‐transformed clutch size and square‐root transformed oviposition preference. Chamber diameter already had a normal distribution so we did not transform it. We then scaled all phenotypic traits (mean = 0 and SD = 1) across treatments prior to our analyses (detailed below) to ensure that our estimates of selection were comparable across traits and with other studies.

Our analysis consisted of four parts. First, we used generalized linear mixed models (GLMM) to quantify selection surfaces—linear and nonlinear relationships between absolute fitness (W) and standardized phenotypic traits (i) of individuals—in each food‐web treatment. Second, we scaled selection surfaces relative to mean fitness ($\bar{W}$) to calculate standardized selection gradients. Third, we used our estimates of directional selection gradients to characterize the slope of the fitness landscape, which we used to quantify the effects of food‐web structure on the trajectory of evolution. Finally, we estimated the curvature of the fitness landscape and used a simulation to explore its effects on the adaptive potential of the gall midge population in the next generation.

#### Selection surfaces

As larval survival was our measure of absolute fitness, we used a GLMM that assumed a binomial error distribution (and logit‐link function). To approximate the selection surface, we modeled larval survival as a function of food‐web treatment as well as linear ($\alpha_{i}$), quadratic ($\alpha_{ii}$), and statistical interactions ($\alpha_{ij}$) between each trait. Note that to obtain valid estimates of linear selection surfaces, we removed nonlinear terms prior to estimating linear relationships (Lande and Arnold 1983). Other approaches have been advocated for approximating selection surfaces (Schluter 1988); however, our approach enables us to calculate selection gradients, and thus is more appropriate for approximating the fitness landscape (Arnold 2003). To account for the nonindependence of clutch size (measured at gall level) and oviposition preference (measured at plant level) as well as any independent effects of willow genotype on larval survival, we modeled gall identity nested within plant identity nested within genotype identity as random effects. Although statistical models with random effects are not common in analyses of natural selection, we think that modeling random effects can mitigate biased estimates of selection due to environmental covariances between traits and fitness (Rausher 1992). As our end goal was to characterize the relationship between mean trait values and mean fitness (fitness landscape), we assumed the mean value of our random effects (i.e., setting them to zero) when calculating selection surfaces. We then used parametric bootstrapping (1000 replicates) to estimate the effect of food‐web treatment on larval survival as well as selection surfaces in each food‐web treatment. To determine whether trait‐fitness relationships differed between food‐web treatments, we calculated the difference in bootstrapped replicates between treatments.

#### Selection gradients

Selection gradients cannot be estimated directly from GLMMs of selection surfaces because the response is in terms of absolute fitness and the coefficients are on a nonlinear scale. For example, the coefficients in the previously described binomial model measure the change in the log‐odds of survival associated with 1SD change in a trait with all other traits held fixed at their mean. Therefore, we used the method developed by Janzen and Stern (1998) to translate selection surfaces from the above model into the scale of relative fitness to calculate directional ($\beta_{i}$), quadratic ($\gamma_{ii}$), and correlational ($\gamma_{ij}$) selection gradients. Briefly, this method calculates the average gradient of selection surfaces by multiplying the average of W(z)[1−W(z)] by each regression coefficient (e.g., $\alpha_{i}$, $\alpha_{ii}$, or $\alpha_{ij}$). We then divided this average gradient by the mean fitness ($\bar{W}$) to put it on the scale of relative fitness ($w=W/\bar{W}$), and thus interpretable as a selection gradient. We estimated selection gradients separately for each food‐web treatment. We also determined whether selection gradients differed between food‐web treatments by calculating the difference in bootstrapped replicates between treatments. Note that we doubled all quadratic terms prior to calculating selection gradients to put them on the same scale as estimates of directional and correlational selection (Stinchcombe et al. 2008).

#### Evolutionary trajectory

The effect of selection on the trajectory of evolution is determined by the slope of the fitness landscape, which in our study corresponds to

$$\text{Slope}=\left(\begin{array}{c} \beta_{\text{Diam}}\\ \beta_{\text{Clutch}}\\ \beta_{\text{Pref}} \end{array}\right),$$

where each $\beta_{i}$ corresponds to the directional selection gradient acting on each trait. By comparing selection gradients in each food‐web treatment (i.e., 95% CI does not overlap zero), we can infer the effect of food‐web structure on the trajectory of phenotypic evolution.

#### Adaptive potential

The indirect effects of selection on the G‐matrix (ΔG=GCG) are governed by the curvature of the fitness landscape ($C=\gamma -\beta \beta^{T}$), which in our study corresponds to

$$\begin{array}{ccc} \text{Curvature} & = & \left(\begin{array}{ccc} \gamma_{\text{Diam:Diam}} & & \\ \gamma_{\text{Clutch:Diam}} & \gamma_{\text{Clutch:Clutch}} & \\ \gamma_{\text{Pref:Diam}} & \gamma_{\text{Pref:Clutch}} & \gamma_{\text{Pref:Pref}} \end{array}\right)\\ & & -\left(\begin{array}{ccc} \beta_{\text{Diam}}\beta_{\text{Diam}} & & \\ \beta_{\text{Clutch}}\beta_{\text{Diam}} & \beta_{\text{Clutch}}\beta_{\text{Clutch}} & \\ \beta_{\text{Pref}}\beta_{\text{Diam}} & \beta_{\text{Pref}}\beta_{\text{Clutch}} & \beta_{\text{Pref}}\beta_{\text{Pref}} \end{array}\right), \end{array}$$

where each $\gamma_{ii}$ (diagonal) corresponds to the quadratic selection gradient acting on a trait, and each $\gamma_{ij}$ (off‐diagonal) corresponds to the correlational selection gradient acting on a particular trait combination. Note that we omitted the upper triangle of each matrix for clarity because it is simply the reflection of the lower triangle. Subtracting these two matrices results in the curvature matrix of the fitness landscape:

$$\text{Curvature}=\left(\begin{array}{ccc} C_{\text{Diam:Diam}} & & \\ C_{\text{Clutch:Diam}} & C_{\text{Clutch:Clutch}} & \\ C_{\text{Pref:Diam}} & C_{\text{Pref:Clutch}} & C_{\text{Pref:Pref}} \end{array}\right),$$

where each $C_{ii}$ (diagonal) represents the effect of selection on the additive genetic variance in a trait, and each $C_{ij}$ (off‐diagonal) represents the effect of selection on the additive genetic covariance between a particular trait combination. We used bootstrapped values of each selection gradient to estimate the curvature of each component of the matrix and its associated 95% CI. We also used this information to determine whether the curvature of each component differed between our food‐web treatments.

Knowledge of the curvature matrix alone gives an incomplete picture of its indirect effect on adaptive potential. This is because the adaptive potential of a population is ultimately determined by the structure of its G‐matrix, and therefore also depends on its structure before selection. Although we do not know the underlying G‐matrix for the traits we measured in this experiment, we can still gain insight to how our food‐web treatment would alter genetic constraints more generally. Specifically, we calculated how our best estimate of the curvature matrix (mean values) in each treatment changed the structure of $10^{4}$ random G‐matrices for the next generation. We restricted the range of additive genetic variance ($V_{G}$) for each element in these G‐matrices to between 0 and 0.5 to reflect typical ranges in narrow‐sense heritability values ($h^{2}$; note that *h* ^2^ $=V_{G}$ when the phenotypic variance is scaled to 1). Note that this analysis assumes that the effects of recombination and mutation on the G‐matrix are much smaller than the effects of selection, which appears to be a reasonable assumption over short time scales (Arnold et al. 2008).

The G‐matrix itself is a complex structure, but has a clear theoretical link to the adaptive potential, or evolvability of phenotypic traits (Hansen and Houle 2008). Evolvability measures the ability for a trait to evolve toward a given direction of selection (Hansen and Houle 2008). In the absence of knowledge about the direction of selection that a population will actually experience in the next generation, we can compute its average evolvability over random directional selection gradients (Hansen and Houle 2008; Melo et al. 2015). By computing the average evolvability (here, we used 1000 random βs) for each of $10^{4}$ random G‐matrices, we can then assess how changes in the curvature matrix alter the adaptive potential of the associated traits. We report the distribution of these effect sizes, rather than conduct a statistical test, because the number of replicates in a simulation can be arbitrarily high, thus making any effect size statistically significant (White et al. 2014).

### ADJUSTING FOR BIASED MEASUREMENTS OF SELECTION

Rather than imposing selection, parasitoids may influence the expression of herbivore traits, which could bias measurements of selection. In our system, it was plausible that parasitoids may influence chamber diameter by altering larval feeding behavior or killing larvae before they complete their development. To estimate this potential bias, we subset our data to only include galls where there was variation in larval survival within the same gall (i.e., 1 > survival > 0). If we assume that larvae within each gall should have similar chamber diameters because they come from the same clutch and experience the same local environment (an assumption our data supports: gall identity explains 54% of the variance in chamber diameter), then the relationship between chamber diameter and larval survival in this data subset represents the effect of parasitism on trait expression (i.e., bias). We used a GLMM with the same structure as described previously except that we modeled only a linear relationship between chamber diameter and larval survival ($\alpha_{\text{Diam}}$). We detected a positive bias in both food‐web treatments (original $\alpha_{\text{Diam}}$= 0.36 [0.05, 0.67]; removal $\alpha_{\text{Diam}}$= 0.42 [0.01, 0.82]), indicating that unadjusted relationships would overestimate the strength of selection on chamber diameter. To account for this bias, we subtracted our mean estimates of bias from our estimates with the full dataset prior to calculating chamber diameter's selection surface and directional selection gradient.

### MEASURING SELECTION ON EGG PARASITOIDS

Once parasitized, the gall phenotype becomes the phenotype of the egg parasitoid. This phenotype may influence the egg parasitoid's survival in the face of larval parasitoids, and thus experiences selection. Our food‐web manipulation allows us to measure selection imposed by larval parasitoids on the phenotype of egg parasitoids. Using the same models as described above, we substituted egg parasitism as our response variable to quantify selection surfaces and selection gradients acting on the egg parasitoid. Note that we cannot test the effect of food‐web structure on the egg parasitoid's fitness landscape—we can only estimate selection imposed by larval parasitoids. This comparison is still useful though in determining the extent to which the loss of consumers may have indirect evolutionary effects by altering selection on multiple interacting populations.

All analyses and visualizations were conducted in R (R Core Team 2018). Unless otherwise noted, we report mean estimates of selection surfaces and selection gradients with 95% confidence intervals in brackets. Note that for visualizing the fitness landscape we restrict trait axes to ±1SD of the mean trait value. This emphasizes the fact that we can only reliably estimate the shape of the fitness landscape near the mean phenotype of the population (Arnold et al. 2001). We also plot mean larval survival on a natural log scale to accurately reflect the mathematical definition of the fitness landscape (Arnold 2003). All data and code to reproduce the reported results are publicly available on GitHub (https://github.com/mabarbour/complexity_selection) and have been archived on Zenodo (https://doi.org/10.5281/zenodo.3706794).

## Results

### CONSUMER REMOVAL CONSTRAINS THE EVOLUTIONARY TRAJECTORY OF GALL MIDGES

**Figure 2:**
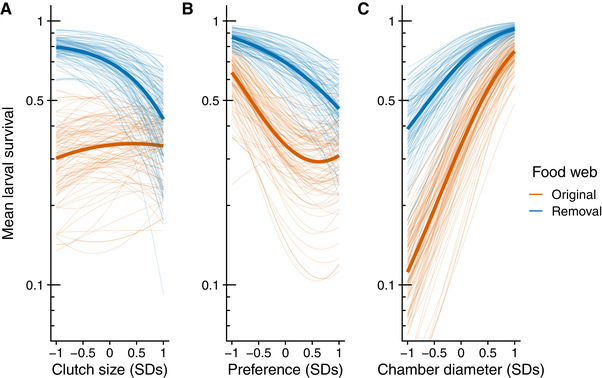
Adaptive landscape of gall midge phenotypes in the original food web and with the removal of larval parasitoids. Each panel corresponds to a different phenotypic trait: clutch size (A); oviposition preference (B); and chamber diameter (C). Bold lines represent selection experienced in the original (orange) and removal (blue) food webs. Thin lines represent bootstrapped replicates to show the uncertainty in selection. For clarity, we only display 100 bootstraps even though inferences are based on 1000 replicates. Note that mean larval survival is plotted on a natural log scale to reflect the mathematical definition of the fitness landscape.

We found that the removal of larval parasitoids increased the number of gall midge traits under directional selection (3 of 3) relative to the original food web (1 of 3) (Table 1). For example, we observed directional selection for smaller clutch sizes when we removed larval parasitoids, but there was no evidence of selection acting on this trait in the original food web (Fig. 2A). This absence of selection appeared to be a result of conflicting selection pressures imposed by each guild of parasitoids. Specifically, when we subset our data to focus on differences between parasitoid guilds, we found that larval parasitoids actually impose directional selection for larger clutch sizes (larval parasitoids $\beta_{\text{Clutch}}$= 0.13 [0.04, 0.24]). This conflicting selection is likely due to trait differences between guilds, as larger clutches may be easier targets for egg parasitoids to find, but the more complex gall structure may be more difficult for larval parasitoids to oviposit through.

**Table 1:** Standardized selection gradients acting on gall midges in the original food web and with the removal of larval parasitoids

Selection gradient	Original	Removal	Contrast (Original–Removal)
$\beta_{\text{Diam}}$	**0.34 [ 0.22, 0.48]**	**0.21 [ 0.12, 0.31]**	**0.14 [ 0, 0.27]**
$\beta_{\text{Clutch}}$	0.06 [ −0.05, 0.17]	**−0.09 [ −0.17, −0.01]**	**0.15 [ 0.03, 0.29]**
$\beta_{\text{Pref}}$	−0.13 [ −0.29, 0.05]	**−0.16 [ −0.26, −0.06]**	0.03 [ −0.15, 0.21]
$\gamma_{\text{Diam:Diam}}$	0.13 [ −0.06, 0.33]	0.1 [ −0.02, 0.23]	0.03 [ −0.2, 0.27]
$\gamma_{\text{Clutch:Clutch}}$	−0.05 [ −0.27, 0.18]	−0.11 [ −0.28, 0.03]	0.06 [ −0.2, 0.32]
$\gamma_{\text{Pref:Pref}}$	**0.34 [ 0.07, 0.63]**	0.02 [ −0.15, 0.18]	**0.32 [ 0.01, 0.64]**
$\gamma_{\text{Diam:Clutch}}$	−0.04 [ −0.16, 0.08]	−0.07 [ −0.15, 0.02]	0.02 [ −0.12, 0.17]
$\gamma_{\text{Diam:Pref}}$	−0.13 [ −0.29, 0.02]	−0.02 [ −0.1, 0.07]	−0.12 [ −0.3, 0.05]
$\gamma_{\text{Clutch:Pref}}$	0.03 [ −0.1, 0.18]	0 [ −0.07, 0.07]	0.03 [ −0.12, 0.18]

*Note*: Values in brackets represent 95% confidence intervals. Bold values indicate that the 95% CI does not overlap zero. $\beta_{\text{Diam}}$ has been adjusted for bias.

We also observed clear evidence of directional selection for midges to avoid ovipositing on plants with high densities of conspecifics when we removed larval parasitoids (Fig. 2B); however, the overall magnitude of selection did not differ between treatments (Table 1). Still, there was no clear evidence of directional selection on oviposition in the original food web (Table 1). Chamber diameter experienced positive directional selection in both food‐web treatments (Fig. 2C). Although the magnitude of selection on chamber diameter was relatively higher in the original food web (Table 1), this was not due to any difference in the relationship between chamber diameter and survival (selection surfaces: contrast $\alpha_{\text{Diam}}$= 0.04 [–0.5, 0.55]), but was simply a consequence of the (expected) lower survival of gall midges in the original food web (contrast $\bar{W}$= 0.27 [0.11, 0.42]). We expect this difference to be transient though, because egg parasitoids would increase in abundance once they are released from intraguild predation, which would make the strength of selection on gall diameter comparable to the original food web (if removal $\bar{W}$=original $\bar{W}$, then contrast $\beta_{\text{Diam}}$= −0.06 [−0.2, 0.1]). It is worth noting that positive selection on chamber diameter in both treatments was unexpected. For example, the fact that larger galls provide more of a refuge from larval parasitoids makes sense because they attack after the gall is formed; however, egg parasitoids attack prior to gall formation, which suggests that chamber diameter is either directly related to survival or strongly correlated with an unmeasured trait that is under selection (e.g., immune response).

**Figure 3:**
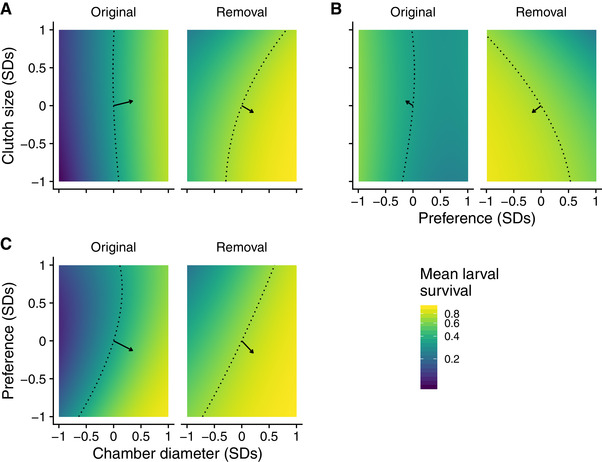
Two‐dimensional view of fitness landscapes of gall midge phenotypes in the original food web and with the removal of larval parasitoids. Each panel corresponds to a different combination of phenotypic traits: clutch size and chamber diameter (A); clutch size and oviposition preference (B); oviposition preference and chamber diameter (C). Arrows represent mean estimates of directional selection gradients, while dotted lines represent predicted larval survival of the mean phenotype in each food‐web treatment. Note that arrows point more toward a corner of the fitness landscape for each combination of traits with the removal of larval parasitoids compared to the original food web. This indicates that the removal of consumers more strongly favored a specific combination of traits. Note that mean larval survival is plotted on a natural log scale to reflect the mathematical definition of the fitness landscape.

To visualize the multivariate effects of natural selection, we plotted the fitness landscape for each trait combination in each treatment (Fig. 3). The arrows in Figure 3 represent mean estimates of directional selection gradients, while the dotted lines represent predicted survival of the mean phenotype in each food‐web treatment. Note that arrows point more toward a corner of the fitness landscape for each combination of traits with the removal of larval parasitoids compared to the original food web. This indicates that the removal of consumers more strongly favored a specific combination of traits, rather than allowing for multiple trait combinations to have comparable fitness.

### CONSUMER REMOVAL CONSTRAINS THE ADAPTIVE POTENTIAL OF GALL MIDGES

The curvature of the fitness landscape indirectly affects adaptive potential and is influenced by directional, quadratic, and correlational selection gradients. There was no clear evidence of correlational selection for any combination of traits in either food‐web treatment (Table 1). Similarly, there was no clear evidence of quadratic selection on chamber diameter or clutch size in either food‐web treatment (Table 1). In contrast, our food‐web treatment did alter quadratic selection acting on oviposition preference (Table 1). In particular, we observed disruptive selection in the original food web, with selection favoring females that either avoided high densities (<0.5 SD above mean density), or if gall densities were high enough (>0.5 SD above mean), then selection favored females that were attracted to high densities (Fig. 2B). This nonlinear relationship was partly due to a trend for disruptive selection imposed by larval parasitoids ($\gamma_{\text{Pref:Pref}}$= 0.18 [−0.02, 0.42]), but was also magnified by the lower average survival in the original food web. This was likely a result of larval parasitoids imposing greater mortality on egg parasitoids at high gall midge densities (see “Selection on egg parasitoids” section), which would act to increase the relative fitness of gall midges at high densities.

Using our estimates of directional ($\beta_{i}$), quadratic ($\gamma_{ii}$), and correlation selection ($\gamma_{ij}$), we calculated the curvature ($C=\gamma -\beta \beta^{T}$) of the fitness landscape in each food‐web treatment.

$$C=\left(\begin{array}{ccc} C_{\text{Diam:Diam}} & & \\ C_{\text{Clutch:Diam}} & C_{\text{Clutch:Clutch}} & \\ C_{\text{Pref:Diam}} & C_{\text{Pref:Clutch}} & C_{\text{Pref:Pref}} \end{array}\right)$$

Of the different components of the curvature matrix, we found that only the curvature of oviposition preference differed between food‐web treatments. Specifically, selection in the removal food web acted to decrease the additive genetic variance in preference relative to the original food web.

$$C_{\text{Original}}=\left(\begin{array}{ccc} -0.05\;[-0.15,0.05] & & \\ -0.06\;[-0.18,0.06] & -0.03\;[-0.14,0.09] & \\ -0.09\;[-0.25,0.06] & 0.04\;[-0.1,0.18] & \\ & 0.15\;[0.02,0.3] \end{array}\right)$$

$$C_{\text{Removal}}=\left(\begin{array}{ccc} 0.01\;[-0.05,0.07] & & \\ -0.05\;[-0.13,0.03] & -0.06\;[-0.15,0.01] & \\ 0.02\;[-0.07,0.1] & -0.01\;[-0.09,0.06] & \\ & -0.02\;[-0.1,0.06] \end{array}\right)$$

$$C_{\text{Removal-Original}}=\left(\begin{array}{ccc} 0.06\;[-0.06,0.18] & & \\ 0.02\;[-0.13,0.16] & -0.03\;[-0.16,0.1] & \\ 0.11\;[-0.06,0.29] & -0.05\;[-0.21,0.1] & \\ & -0.17\;[-0.33,-0.01] \end{array}\right)$$

**Figure 4:**
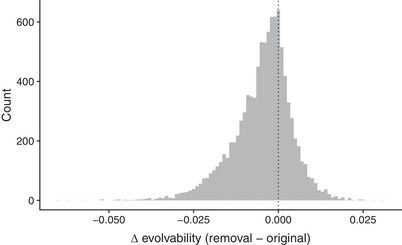
Change in average evolvability for 10,000 random *G*‐matrices using our best (mean) estimate of the curvature matrix for each food‐web treatment. We found that the curvature of the removal food web decreased evolvability in 71% of the *G*‐matrices (i.e., the change in evolvability was negative for 71% of the simulations), suggesting that the removal of consumers tended to decrease adaptive potential of traits in our study.

Interestingly, when we translate the effect of the curvature matrix onto adaptive potential in the next generation, we see that the removal of consumers had a general tendency to decrease evolvability (Fig. 4). Specifically, the removal food web decreased the average evolvability of 71% of the random G‐matrices in our simulation. If anything, we expect that this underestimates the true effect of our removal treatment. For example, if we assume egg parasitoids would eventually impose similar impact on mean fitness once they are released from intraguild predation (i.e., removal $\bar{W}$ = original $\bar{W}$), then the removal food web decreases the average evolvability in 78% of the G‐matrix scenarios.

### SELECTION ON EGG PARASITOIDS

**Figure 5:**
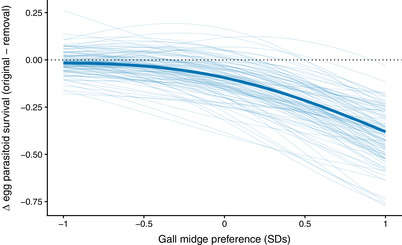
Selection imposed by larval parasitoids on egg parasitoids (*Platygaster* sp.). The bold line represents the average difference in the probability of observing the egg parasitoid (original minus removal of larval parastioids) as a function of gall midge oviposition preference. Thin lines represent bootstrapped replicates to show the uncertainty in selection. For clarity, we only display 100 bootstraps even though inferences are based on 1000 replicates. The decrease in the probability of observing egg parasitoids at high gall‐midge densities indicate that larval parasitoids impose nonlinear selection on egg parasitoids.

The removal of larval parasitoids not only had direct effects on gall midge fitness, but also imposed indirect effects that would be felt in the next generation. For example, the removal of larval parasitoids altered the relationship between gall midge preference and the probability of observing egg parasitoids ($\gamma_{\text{Pref:Pref}}$ = −0.46 [ −1.07, −0.02], Table S1), such that the impact of larval parasitoids increased nonlinearly with higher gall midge densities (Fig. 5). This may indicate that larval parasitoids switch to target galls that have been parasitized by an egg parasitoid once egg parasitoid densities are high enough. This prey switching behavior would contribute to the disruptive selection we observed on gall midge preference by increasing the relative fitness of gall midges at high densities (Fig. 2B).

## Discussion

We found that the removal of larval parasitoids constrained phenotypic evolution in gall midges in two key ways. First, we observed directional selection on more traits in the absence of larval parasitoids, suggesting greater constraints on the trajectory of phenotypic evolution. Second, removing larval parasitoids altered the curvature of the fitness landscape in such a way that tended to decrease the evolvability of associated traits. Assuming these traits have other ecological functions, then this decrease in evolvability could constrain the gall midge's adaptive potential in the face of novel selection pressures. Our experiment also revealed evidence of indirect selection pressures, suggesting that the loss of consumers may have complex effects on the trajectories of phenotypic evolution. Taken together, our study provides experimental evidence from the field that the loss of consumers may constrain the adaptive potential of remaining populations.

The generality of our results likely depends on the relative abundance and functional differences between consumers in a community. For example, if consumers do not differ from each other, then we do not expect the loss of consumers to modify selective constraints. Also, many consumers may be at too low of abundances to impose selection on their resources. Rank abundance curves (Preston 1948) and the disproportionate number of weak interactions in diverse communities (Paine 1992) support this notion. This logic suggests that we may not have observed the effects we did if we had only removed one larval parasitoid because each species had relatively low abundance (Barbour et al. 2016) and they likely share similar ecological roles. When consumers are functionally different and abundant though, the effect of consumer loss will depend on whether different consumers impose conflicting selection pressures or select for distinct traits. When consumers impose conflicting selection on traits, as in our study and others (Weis and Abrahamson 1985; Abrahamson and Weis 1997; Start and Gilbert 2016; Start et al. 2018), then consumer diversity acts to neutralize selection, which can maintain larger evolvability. On the other hand, different consumers may impose selection on different traits; therefore, a more diverse consumer community may favor a particular combination of traits and increase selective constraints. Examples of this include strong genetic covariances in plant resistance to different insect herbivores (Maddox and Root 1990; Wise 2007; Wise and Rausher 2013), although there are also examples where these covariances are weak (Roche and Fritz 1997; Barbour et al. 2015), or vary from year to year (Johnson and Agrawal 2007). We suggest that gaining predictive insight to the evolutionary consequences of food‐web disassembly requires an understanding of the mechanisms governing the assembly of trophic interactions (Bascompte and Stouffer 2009).

We also found evidence for a general decrease in trait evolvability when we excluded larval parasitoids due to changes in the curvature of the fitness landscape. This result was driven by disruptive selection on gall midge oviposition preference in the original food web, which was likely due to both increases in intraguild predation on egg parasitoids (i.e., prey switching) and the lower mean survival of gall midges. This pattern of selection acts to increase genetic variation in oviposition preference, which in turn enhances the ability of the gall midge population to respond to uncertain selection pressures in the next generation (i.e., evolvability). This pattern of selection also indicates the possibility of evolutionary bistability, where different initial conditions (e.g., mean oviposition preference) select for different phenotypes of the same species in similar environments (Landi et al. 2018b). Thus, this pattern of selection may contribute to genetic and phenotypic diversity both within and among populations. Current theory, however, often assumes genetic variances and covariances remain constant over time and space rather than dynamically changing with the network context (McPeek 2017; Guimarães et al. 2017; Medeiros et al. 2018). Our empirical results highlight the need to explore the evolutionary consequences of not only direct effects of selection, but indirect effects on genetic constraints that emerge in a network of interacting species.

An important caveat of our study is that we did not do a factorial manipulation of both parasitoid guilds, making it difficult to conclude whether our results would change if we manipulated the presence/absence of the dominant egg parasitoid. If we assume that higher order interactions (Levine et al. 2017) are weak between parasitoid guilds, then we can gain insight to how the loss of the egg parasitoid would alter selection by isolating the contribution of larval parasitoids to selection in our original food‐web treatment. When we do this, we see the same qualitative effects as we do when we removed larval parasitoids. For example, we see clear evidence of all three traits being under directional selection (i.e., greater constraints on the trajectory of evolution, Table S2) as well as a decrease, albeit smaller, in trait evolvability under different G‐matrix scenarios (57%, Fig. S1). This suggests that our results could be robust to this caveat, which was simply not possible to manipulate given the biology of our system (see “Manipulating Food‐web Structure” section for explanation).

Our results suggest that the loss of consumers may not only directly affect connected species, but also result in indirect evolutionary effects. In our study, this indirect effect arises from egg parasitoids being released from intraguild predation when we excluded larval parasitoids. This release occurs more on trees with high larval densities, which could intensify future selection on gall midge oviposition preference. This suggests that the loss of larval parasitoids would likely impose even greater constraints on the trajectory of evolution than our current estimates indicate. Interestingly, this increase in the strength of selection on gall midges may reduce their densities, which would weaken selection imposed on willows by the gall midge through an evolutionary trophic cascade. A growing number of experiments over the past two decades have demonstrated the presence and potential importance of indirect evolutionary effects that emerge in ecological communities (Pilson 1996; Juenger and Bergelson 1998; Stinchcombe and Rausher 2001; Lankau and Strauss 2007; Walsh and Reznick 2008, 2010; terHorst 2010; Sahli and Conner 2011; Lau 2012; terHorst et al. 2015; Schiestl et al. 2018; Start et al. 2019). If indirect evolutionary effects are common (Miller and Travis 1996; Walsh 2013; Guimarães et al. 2017), then predicting evolutionary trajectories resulting from the loss of consumers will require evolutionary studies to explicitly account for the ecological networks that species are embedded in.

Our study gives insight to how the loss of consumers alters natural selection, and in turn the evolutionary trajectory and adaptive potential of remaining populations. In particular, it hints at a potential insidious effect of local extinctions that compromises the robustness of remaining populations to future environmental change. Our work also highlights some key challenges for predicting phenotypic evolution in rapidly changing communities. For example, many theoretical models of ecoevolutionary dynamics focus on phenotypic change in a single trait (but see Brown et al. 2007; Leimar 2009), yet our results highlight that the number of traits under selection may change with the network context. Importantly, we found that different species/guilds imposed different selection pressures. Knowing these hidden selection pressures is critical for prediction because the trajectory of evolution will depend on the nature of change in the ecological community. We expect that a continued integration of fitness landscapes and ecological networks will enhance our ability to predict the evolutionary consequences of changes in ecological communities.

Associate Editor: R. Snook

## Supporting information


**Table S1**: Standardized selection gradients acting on egg parasitoids (*Platygaster* sp.)
**Table S2**: Standardized selection gradients imposed by larval parasitoids on gall midges in the original food web.
**Figure S1**: Change in average evolvability for 10,000 random G‐matrices using our best (mean) estimate of the curvature matrix for selection in the absence of egg parasitoids vs. the original food web.

## References

[evl3170-bib-0001] Abrahamson, W. G. , and A. E. Weis . 1997 Evolutionary ecology across three trophic levels: goldenrods, gallmakers, and natural enemies Princeton Univ. Press, Princeton, NJ.

[evl3170-bib-0002] Abrams, P. A. 2000 The evolution of predator‐prey interactions: theory and evidence. Annu. Rev. Ecol. Evol. 31:79–105.

[evl3170-bib-0003] Arnold, S. J. 1992 Constraints on phenotypic evolution. Am. Nat. 140:S85–S107. 19426028 10.1086/285398

[evl3170-bib-0004] Arnold, S. J. 2003 Performance surfaces and adaptive landscapes. Integr. Comp. Biol. 43:367–375. 21680445 10.1093/icb/43.3.367

[evl3170-bib-0005] Arnold, S. J. , and M. J. Wade . 1984a On the measurement of natural and sexual selection: applications. Evolution 38:720–734. 28555830 10.1111/j.1558-5646.1984.tb00345.x

[evl3170-bib-0006] Arnold, S. J. 1984b On the measurement of natural and sexual selection: theory. Evolution 38:709–719. 28555816 10.1111/j.1558-5646.1984.tb00344.x

[evl3170-bib-0007] Arnold, S. J. , M. E. Pfrender , and A. G. Jones . 2001 The adaptive landscape as a conceptual bridge between micro‐ and macroevolution. Genetica 112:9–32. 11838790

[evl3170-bib-0008] Arnold, S. J. , R. Bürger , P. A. Hohenlohe , B. C. Ajie , and A. G. Jones . 2008 Understanding the evolution and stability of the g‐matrix. Evolution 62:2451–2461. 18973631 10.1111/j.1558-5646.2008.00472.x PMC3229175

[evl3170-bib-0009] Barbour, M. A. , M. A. Rodriguez‐Cabal , E. T. Wu , R. Julkunen‐Tiitto , C. E. Ritland , A. E. Miscampbell , et al. 2015 Multiple plant traits shape the genetic basis of herbivore community assembly. Funct. Ecol. 29:995–1006.

[evl3170-bib-0010] Barbour, M. A. , M. A. Fortuna , J. Bascompte , J. R. Nicholson , R. Julkunen‐Tiitto , E. S. Jules , et al. 2016 Genetic specificity of a plant–insect food web: implications for linking genetic variation to network complexity. Proc. Natl. Acad. Sci. USA 113:2128–2133. 26858398 10.1073/pnas.1513633113 PMC4776535

[evl3170-bib-0011] Bascompte, J. and P. Jordano . 2014 Mutualistic Networks. Princeton University Press http://www.jstor.org/stable/j.ctt5hhnpq.

[evl3170-bib-0012] Bascompte, J. , and D. B. Stouffer . 2009 The assembly and disassembly of ecological networks. Philos. Trans. R. Soc. Lond. B 364:1781–1787. 19451127 10.1098/rstb.2008.0226 PMC2685423

[evl3170-bib-0013] Bolnick, D. I. , and O. L. Lau . 2008 Predictable patterns of disruptive selection in stickleback in postglacial lakes. Am. Nat. 172:1–11. 18452402 10.1086/587805

[evl3170-bib-0014] Brodie, E. D. 1992 Correlational selection for color pattern and antipredator behavior in the garter snake *Thamnophis ordinoides* . Evolution 46:1284–1298. 28568995 10.1111/j.1558-5646.1992.tb01124.x

[evl3170-bib-0015] Bronstein, J. L. , R. Alarcón , and M. Geber . 2006 The evolution of plant‐insect mutualisms. New Phytol. 172:412–428. 17083673 10.1111/j.1469-8137.2006.01864.x

[evl3170-bib-0016] Brown, J. S. , Y. Cohen , and T. L. Vincent . 2007 Adaptive dynamics with vector‐valued strategies. Evol. Ecol. Res. 9:719–756.

[evl3170-bib-0017] Gagné, R. J. 1989 The plant‐feeding gall midges of North America. Cornell Univ. Press, Ithaca, NY.

[evl3170-bib-0018] Grant, P. R. , and B. R. Grant . 1995 Predicting microevolutionary responses to directional selection on heritable variation. Evolution 49:241–251. 28565006 10.1111/j.1558-5646.1995.tb02236.x

[evl3170-bib-0019] Gripenberg, S. , P. J. Mayhew , M. Parnell , and T. Roslin . 2010 A meta‐analysis of preference–performance relationships in phytophagous insects. Ecology Letters 13:383–393. 20100245 10.1111/j.1461-0248.2009.01433.x

[evl3170-bib-0020] Guimarães, P. R., Jr , M. M. Pires , P. Jordano , J. Bascompte , and J. N. Thompson . 2017 Indirect effects drive coevolution in mutualistic networks. Nature 550:511–514. 29045396 10.1038/nature24273

[evl3170-bib-0021] Hansen, T. F. , and D. Houle . 2008 Measuring and comparing evolvability and constraint in multivariate characters. J. Evol. Biol. 21:1201–1219. 18662244 10.1111/j.1420-9101.2008.01573.x

[evl3170-bib-0022] Hawkins, B. A. , H. V. Cornell , and M. E. Hochberg . 1997 Predators, parasitoids, and pathogens as mortality agents in phytophagous insect populations. Ecology 78:2145–2152.

[evl3170-bib-0023] Heath, J. J. , P. Abbot , and J. O. Stireman . 2018 Adaptive divergence in a defense symbiosis driven from the top down. Am Nat. 192:E21–E36. 29897808 10.1086/697446

[evl3170-bib-0024] Hui, C. , H. O. Minoarivelo , and P. Landi . 2018 Modelling coevolution in ecological networks with adaptive dynamics. Math. Methods Appl. Sci. 41:8407–8422.

[evl3170-bib-0025] Janzen, F. J. , and H. S. Stern . 1998 Logistic regression for empirical studies of multivariate selection. Evolution 52:1564–1571. 28565316 10.1111/j.1558-5646.1998.tb02237.x

[evl3170-bib-0026] Johnson, M. T. J. , and A. A. Agrawal . 2007 Covariation and composition of arthropod species across plant genotypes of evening primrose, *Oenothera biennis* . Oikos 116:941–956.

[evl3170-bib-0027] Juenger, T. , and J. Bergelson . 1998 Pairwise versus diffuse natural selection and the multiple herbivores of scarlet gilia, *Ipomopsis aggregata* . Evolution 52:1583–1592. 28565335 10.1111/j.1558-5646.1998.tb02239.x

[evl3170-bib-0028] Lande, R. 1979 Quantitative genetic analysis of multivariate evolution, applied to brain:body size allometry. Evolution 33: 402–416. 28568194 10.1111/j.1558-5646.1979.tb04694.x

[evl3170-bib-0029] Lande, R. , and S. J. Arnold . 1983 The measurement of selection on correlated characters. Evolution 37:1210–1226. 28556011 10.1111/j.1558-5646.1983.tb00236.x

[evl3170-bib-0030] Landi, P. , H. O. Minoarivelo , Å. Brännström , C. Hui , and U. Dieckmann . 2018a Complexity and stability of ecological networks: a review of the theory. Popul. Ecol. 60:319–345.

[evl3170-bib-0031] Landi, P. , J. R. Vonesh , and C. Hui . 2018b Variability in life‐history switch points across and within populations explained by adaptive dynamics. J. R. Soc. Interface 15 10.1098/rsif.2018.0371. PMC6283999 30429260

[evl3170-bib-0032] Lankau, R. A. , and S. Y. Strauss . 2007 Community complexity drives patterns of natural selection on a chemical defense of *Brassica nigra* . Am. Nat. 171:150–161. 10.1086/524959 18197768

[evl3170-bib-0033] Lau, J. A. 2012 Evolutionary indirect effects of biological invasions. Oecologia 170:171–181. 22402621 10.1007/s00442-012-2288-x

[evl3170-bib-0034] Leimar, O. 2009 Multidimensional convergence stability. Evol. Ecol. Res. 11:191–208.

[evl3170-bib-0035] Levine, J. M. , J. Bascompte , P. B. Adler , and S. Allesina . 2017 Beyond pairwise mechanisms of species coexistence in complex communities. Nature 546:56–64. 28569813 10.1038/nature22898

[evl3170-bib-0036] Maddox, G. D. , and R. B. Root . 1990 Structure of the encounter between goldenrod (*Solidago altissima*) and its diverse insect fauna. Ecology 71:2115–2124.

[evl3170-bib-0037] McCann, K. S. 2012. Food webs. Princeton Univ. Press, Princeton, NJ.

[evl3170-bib-0038] McPeek, M. A. 2017 Evolutionary community ecology. Princeton Univ. Press, Princeton, NJ.

[evl3170-bib-0039] Medeiros, L. P. , G. Garcia , J. N. Thompson , and P. R. Guimarães, Jr . 2018 The geographic mosaic of coevolution in mutualistic networks. Proc. Natl. Acad. Sci. USA 115:12017–12022. 30404910 10.1073/pnas.1809088115 PMC6255164

[evl3170-bib-0040] Melo, D. , G. Garcia , A. Hubbe , A. P. Assis , and G. Marroig . 2015 EvolQG—an R package for evolutionary quantitative genetics. F1000Res. 4:925. 27785352 10.12688/f1000research.7082.1 PMC5022708

[evl3170-bib-0041] Miller, T. E. , and J. Travis . 1996 The evolutionary role of indirect effects in communities. Ecology 77:1329–1335.

[evl3170-bib-0042] Paine, R. T. 1992 Food‐web analysis through field measurement of per capita interaction strength. Nature 355:73–75.

[evl3170-bib-0043] Pilson, D. 1996 Two herbivores and constraints on selection for resistance in *Brassica rapa* . Evolution 50:1492–1500. 28565704 10.1111/j.1558-5646.1996.tb03922.x

[evl3170-bib-0044] Preston, F. W. 1948 The commonness, and rarity, of species. Ecology 29:254–283.

[evl3170-bib-0045] R Core Team , 2018 R: a language and environment for statistical computing. R Foundation for Statistical Computing, Vienna, Austria.

[evl3170-bib-0046] Rausher, M. D. 1992 The measurement of selection on quantitative traits: biases due to environmental covariances between traits and fitness. Evolution 46:616–626. 28568666 10.1111/j.1558-5646.1992.tb02070.x

[evl3170-bib-0047] Roche, B. M. , and R. S. Fritz . 1997 Genetics of resistance of *Salix sericea* to a diverse community of herbivores. Evolution 51:1490–1498. 28568638 10.1111/j.1558-5646.1997.tb01472.x

[evl3170-bib-0048] Russo, R. 2006 Field guide to plant galls of California and other western states. Univ. of California Press, Berkeley, CA.

[evl3170-bib-0049] Sahli, H. F. , and J. K. Conner . 2011 Testing for conflicting and nonadditive selection: floral adaptation to multiple pollinators through male and female fitness. Evolution 65:1457–1473. 21521195 10.1111/j.1558-5646.2011.01229.x

[evl3170-bib-0050] Scheffers, B. R. , L. De Meester , T. C. L. Bridge , A. A. Hoffmann , J. M. Pandolfi , R. T. Corlett , et al. 2016 The broad footprint of climate change from genes to biomes to people. Science 354: aaf7671‐7. 27846577 10.1126/science.aaf7671

[evl3170-bib-0051] Schiestl, F. P. , A. Balmer , and D. D. Gervasi . 2018 Real‐time evolution supports a unique trajectory for generalized pollination. Evolution 72:2653–2668. 30257033 10.1111/evo.13611

[evl3170-bib-0052] Schluter, D. 1988 Estimating the form of natural selection on a quantitative trait. Evolution 42:849–861. 28581168 10.1111/j.1558-5646.1988.tb02507.x

[evl3170-bib-0053] Schluter, D. 2000 Ecological character displacement in adaptive radiation. Am. Nat. 156:S4–S16.

[evl3170-bib-0054] Simpson, G. G. 1944 Tempo and mode in evolution. Columbia Univ. Press, New York.

[evl3170-bib-0055] Singer, M. C. 1986 The definition and measurement of oviposition preference in plant‐feeding insects Pp. 65–94 *in* Miller J. R. and Miller T. A., eds. Insect‐plant interactions. Springer, New York, NY.

[evl3170-bib-0056] Start, D. , C. Bonner , A. E. Weis , and B. Gilbert . 2018 Consumer‐resource interactions along urbanization gradients drive natural selection. Evolution 72:1863–1873. 29972241 10.1111/evo.13544

[evl3170-bib-0057] Start, D. , and B. Gilbert . 2016 Host–parasitoid evolution in a metacommunity. Proc. R. Soc. B 283: 20160477. 10.1098/rspb.2016.0477 PMC4892800 27194705

[evl3170-bib-0058] Start, D. , A. E. Weis , and B. Gilbert . 2019 Indirect interactions shape selection in a multi‐species foodweb. Am. Nat. 193:321–330. 30794449 10.1086/701785

[evl3170-bib-0059] Stinchcombe, J. R. , and M. D. Rausher . 2001 Diffuse selection on resistance to deer herbivory in the ivyleaf morning glory, *Ipomoea hederacea* . Am. Nat. 158:376–388. 18707334 10.1086/321990

[evl3170-bib-0060] Stinchcombe, J. R. , A. F. Agrawal , P. A. Hohenlohe , S. J. Arnold , and M. W. Blows . 2008 Estimating nonlinear selection gradients using quadratic regression coefficients: double or nothing? Evolution 62:2435–2440. 18616573 10.1111/j.1558-5646.2008.00449.x

[evl3170-bib-0061] terHorst, C. P. 2010 Evolution in response to direct and indirect ecological effects in pitcher plant inquiline communities. Am. Nat. 176:675–685. 20955011 10.1086/657047

[evl3170-bib-0062] terHorst, C. P. , J. A. Lau , I. A. Cooper , K. R. Keller , R. J. La Rosa , A. M. Royer , et al. 2015 Quantifying nonadditive selection caused by indirect ecological effects. Ecology 96:2360–2369. 26594694 10.1890/14-0619.1

[evl3170-bib-0063] terHorst, C. P. , P. C. Zee , K. D. Heath , T. E. Miller , A. I. Pastore , S. Patel , et al. 2018 Evolution in a community context: trait responses to multiple species interactions. Am. Nat. 191:368–380.

[evl3170-bib-0064] Walsh, M. R. 2013 The evolutionary consequences of indirect effects. Trends Ecol Evol. 28:23–29. 22944194 10.1016/j.tree.2012.08.006

[evl3170-bib-0065] Walsh, M. R. , and D. N. Reznick . 2008 Interactions between the direct and indirect effects of predators determine life history evolution in a killifish. Proc. Natl. Acad. Sci. USA 105:594–599. 18180455 10.1073/pnas.0710051105 PMC2206581

[evl3170-bib-0066] Walsh, M. R. 2010 Influence of the indirect effects of guppies on life‐history evolution in *Rivulus hartii* . Evolution 64:1583–1593. 20015237 10.1111/j.1558-5646.2009.00922.x

[evl3170-bib-0067] Weis, A. E. , and W. G. Abrahamson . 1985 Potential selective pressures by parasitoids on a plant‐herbivore interaction. Ecology 66:1261–1269.

[evl3170-bib-0068] Weis, A. E. , P. W. Price , and M. Lynch . 1983 Selective pressures on clutch size in the gall maker *Asteromyia carbonifera* . Ecology 64:688–695.

[evl3170-bib-0069] White, J. W. , A. Rassweiler , J. F. Samhouri , A. C. Stier , and C. White . 2014 Ecologists should not use statistical significance tests to interpret simulation model results. Oikos 123:385–388.

[evl3170-bib-0070] Wise, M. J. 2007 Evolutionary ecology of resistance to herbivory: an investigation of potential genetic constraints in the multiple‐herbivore community of *Solanum carolinense* . New Phytol. 175:773–784. 17688592 10.1111/j.1469-8137.2007.02143.x

[evl3170-bib-0071] Wise, M. J. , and M. D. Rausher . 2013 Evolution of resistance to a multiple‐herbivore community: genetic correlations, diffuse coevolution, and constraints on the plant's response to selection. Evolution 67:1767–1779. 23730768 10.1111/evo.12061

[evl3170-bib-0072] Wright, S. 1931 Evolution in mendelian populations. Genetics 16:97–159. 17246615 10.1093/genetics/16.2.97 PMC1201091

