# Supplementary material for: Loss of consumers constrains phenotypic evolution in the resulting food web
Source: Evol Lett. 2020 Apr 20;4(3):266–77. doi: 10.1002/evl3.170 (PMC7293086; doi:10.1002/evl3.170)
Supplement: Supplementary file 1 — Table S1: Standardized selection gradients acting on egg parasitoids (Platygaster sp.) Table S2: Standardized selection gradients imposed by larval parasitoids on gall midges in the original food web. Figure S1: Change in average evolvability for 10,000 random G‐matrices using our best (mean) estimate of the curvature matrix for selection in the absence of egg parasitoids vs. the original food web. [file EVL3-4-266-s001.pdf]

# Supplementary Material:

# Loss of consumers constrains phenotypic evolution in the resulting food web

Matthew A. Barbour[1,2,*], Christopher J. Greyson-Gaito[2,3], Arezoo Sootodeh[2], Brendan Locke[4], Jordi Bascompte[1]

1. University of Zurich, Department of Evolutionary Biology and Environmental Studies, Zurich, 8057 ZH, Switzerland;

2. University of British Columbia, Department of Zoology, Vancouver, BC V6T 1Z4, Canada;

3. University of Guelph, Department of Integrative Biology, Guelph, ON N1G 2W1, Canada;

4. Humboldt State University, Department of Biological Sciences, Arcata, CA 95521, USA.

* Corresponding author; e-mail: matthew.barbour@ieu.uzh.ch

Table S1: Standardized selection gradients acting on egg parasitoids (*Platygaster* sp.)

| **Selection gradient** | **Contrast = Original - Removal** |
|---|---|
| $\beta_{\text{Diam}}$ | -0.03 [ -0.3, 0.25] |
| $\beta_{\text{Clutch}}$ | 0.07 [ -0.26, 0.39] |
| $\beta_{\text{Pref}}$ | -0.25 [ -0.64, 0.09] |
| $\gamma_{\text{Diam:Diam}}$ | -0.05 [ -0.43, 0.33] |
| $\gamma_{\text{Clutch:Clutch}}$ | -0.21 [ -0.68, 0.26] |
| $\gamma_{\text{Pref:Pref}}$ | **-0.46 [ -1.07, -0.02]** |
| $\gamma_{\text{Diam:Clutch}}$ | 0 [ -0.29, 0.27] |
| $\gamma_{\text{Diam:Pref}}$ | 0.25 [ -0.04, 0.6] |
| $\gamma_{\text{Clutch:Pref}}$ | -0.18 [ -0.52, 0.12] |

Note: Values in brackets represent 95% confidence intervals. Bold values indicate that the 95% CI does not overlap zero.

$\beta_{\text{Diam}}$ has been adjusted for bias.

Table S2: Standardized selection gradients imposed by larval parasitoids on gall midges in the original food web. This gives insight to selection acting on gall midges in the absence of the dominant egg parasitoid.

| **Selection gradient** | **Larval Parasitoids** |
|---|---|
| $\beta_{\text{Diam}}$ | **0.23 [ 0.13, 0.36]** |
| $\beta_{\text{Clutch}}$ | **0.13 [ 0.04, 0.24]** |
| $\beta_{\text{Pref}}$ | **-0.17 [ -0.34, -0.03]** |
| $\gamma_{\text{Diam:Diam}}$ | 0.06 [ -0.07, 0.2] |
| $\gamma_{\text{Clutch:Clutch}}$ | 0.01 [ -0.14, 0.15] |
| $\gamma_{\text{Pref:Pref}}$ | 0.18 [ -0.02, 0.42] |
| $\gamma_{\text{Diam:Clutch}}$ | 0.02 [ -0.07, 0.11] |
| $\gamma_{\text{Diam:Pref}}$ | -0.05 [ -0.18, 0.06] |
| $\gamma_{\text{Clutch:Pref}}$ | -0.04 [ -0.14, 0.06] |

Note: Values in brackets represent 95% confidence intervals. Bold values indicate that the 95% CI does not overlap zero.

$\beta_{\text{Diam}}$ has been adjusted for bias.

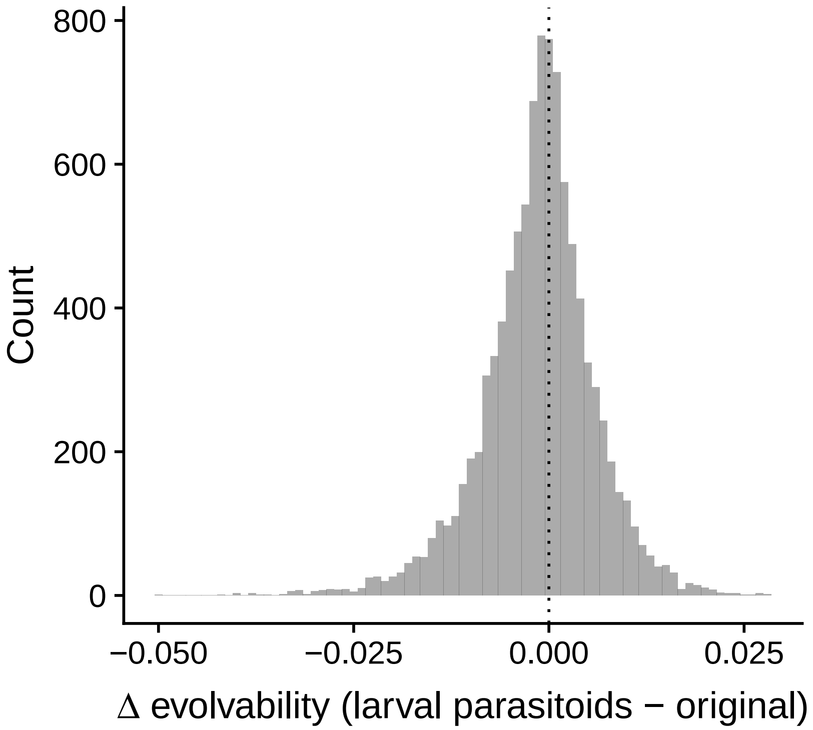


Figure S1: Change in average evolvability for 10,000 random G-matrices using our best (mean) estimate of the curvature matrix for selection in the absence of egg parasitoids vs. the original food web. We found that the curvature imposed by the simulated removal of egg parasitoids decreased evolvability in 57% of the G-matrices (i.e. the change in evolvability was negative for 57% of the simulations), which is smaller in magnitude, but in the same direction, as the effects of removing larval parasitoids.
